# Kidney-Related Function of Mitochondrial Protein Mitoregulin

**DOI:** 10.3390/ijms24109106

**Published:** 2023-05-22

**Authors:** Olga A. Averina, Oleg A. Permyakov, Mariia A. Emelianova, Ekaterina A. Guseva, Olga O. Grigoryeva, Maxim L. Lovat, Anna E. Egorova, Andrei V. Grinchenko, Vadim V. Kumeiko, Maria V. Marey, Vasily N. Manskikh, Olga A. Dontsova, Mikhail Y. Vyssokikh, Petr V. Sergiev

**Affiliations:** 1 Institute of Functional Genomics, Lomonosov Moscow State University, 119992 Moscow, Russia; averina.olga.msu@gmail.com (O.A.A.); norad_m@mail.ru (O.A.P.); grig_forever@mail.ru (O.O.G.); 2 Belozersky Institute of Physico-Chemical Biology, Lomonosov Moscow State University, 119992 Moscow, Russia; lovat@mail.ru (M.L.L.); manskikh@mail.ru (V.N.M.); olga.a.dontsova@gmail.com (O.A.D.); 3 Center for Life Sciences, Skolkovo Institute of Science and Technology, 143025 Moscow, Russia; mshep98@mail.ru (M.A.E.); eguseva98@mail.ru (E.A.G.); 4 Institute of Mitoengineering MSU, 119992 Moscow, Russia; 5 Institute of Life Sciences and Biomedicine, Far Eastern Federal University, 690922 Vladivostok, Russia; bioanna95@list.ru (A.E.E.); kumeyko.vv@dvfu.ru (V.V.K.); 6 A.V. Zhirmunsky National Scientific Center of Marine Biology, 690041 Vladivostok, Russia; grishagrin@mail.ru; 7 National Medical Research Center for Obstetrics, Gynecology and Perinatology Named after Academician V.I.Kulakov, 117198 Moscow, Russia; m_marey@oparina4.ru; 8 Department of Chemistry, Lomonosov Moscow State University, 119991 Moscow, Russia; 9 Shemyakin-Ovchinnikov Institute of Bioorganic Chemistry, Russian Academy of Sciences, 119992 Moscow, Russia

**Keywords:** kidney, small peptide, oxidative phosphorylation, mitochondria, metabolism, mitoregulin

## Abstract

A small protein, Mitoregulin (Mtln), localizes in mitochondria and contributes to oxidative phosphorylation and fatty acid metabolism. *Mtln* knockout mice develop obesity on a high-fat diet, demonstrating elevated cardiolipin damage and suboptimal creatine kinase oligomerization in muscle tissue. Kidneys heavily depend on the oxidative phosphorylation in mitochondria. Here we report kidney-related phenotypes in aged *Mtln* knockout mice. Similar to *Mtln* knockout mice muscle mitochondria, those of the kidney demonstrate a decreased respiratory complex I activity and excessive cardiolipin damage. Aged male mice carrying *Mtln* knockout demonstrated an increased frequency of renal proximal tubules’ degeneration. At the same time, a decreased glomerular filtration rate has been more frequently detected in aged female mice devoid of Mtln. An amount of Mtln partner protein, Cyb5r3, is drastically decreased in the kidneys of *Mtln* knockout mice.

## 1. Introduction

Small proteins encoded in short open reading frames are an emerging biomolecule largely overlooked in the past [1,2,3,4]. Out of slightly over 1000 proteins that compose a mitochondrial proteome, more than 5% are small [5], which is below the length limit of 100 amino acids. Among them are newly discovered peptides, such as MIEF1, a component of mitoribosome [6,7], RP11_469A15.2 gene product interacting with cytochrome oxidase complex [8], BRAWNIN regulator of cytochrome c oxidase [9] and PIGBOS mediating mitochondria to the endoplasmic reticulum (ER) contacts [10] (see [11] for a recent review). One of the small mitochondrial peptides identified recently is the mitochondrial peptide Mtln [12,13,14]. Its inactivation in cell lines and mice leads to respiratory chain defects [12,13,14,15] and dysregulation of fatty acid metabolism [12,13,14,16]. More recently, Mtln function in mitochondria-ER cross-talk was discovered [17]. Mice with inactivated *Mtln* genes demonstrate several muscle-related phenotypes, such as lower performance on a treadmill or rotarod [14,18], reduction of the grip strength [18,19,20], smaller myofibrils [18,19], increased cardiolipin damage [20] and several respiratory phenotypes [13,14,18]. Another tissue found to be affected by *Mtln* inactivation is fat tissue. *Mtln* knockout in adipocytes resulted in triacylglycerol accumulation [15], while inactivation of the *Mtln* gene in mice likewise resulted in excessive fat accumulation on a high-fat diet [16]. 

However, muscles and fat are not the only tissues that depend heavily on mitochondrial respiration. The kidney is one of the major energy-consuming organs whose solute pumps rely on mitochondrial oxidative phosphorylation as a source of ATP [21]. Several well-known mitochondrial dysfunctions, such as MELAS (mitochondrial encephalomyopathy, lactic acidosis, and stroke-like symptoms), MERRF (myoclonus, epilepsy with ragged red fibers), Pearson, Kearns–Sayre, and Leigh syndromes have kidney-related manifestations [22]. Among the renal phenotypes of mitochondrial disorders are tubulointerstitial nephritis, focal segmental glomerulosclerosis, renal tubular acidosis, proximal and distal tubulopathy, methylmalonic aciduria, cystathioninuria, and progressive renal failure (see [22,23] for reviews). Mitochondrial peptides were recently demonstrated to be important molecular markers of chronic kidney disease. Humanin, a 24-amino acid peptide encoded in the mitochondrial 16S rRNA gene, was found at elevated levels in the serum upon chronic kidney disease, while levels of MOTS-c, a 16-amino acid peptide whose open reading frame is found in the mitochondrial 12S rRNA, were decreased upon the same pathologic condition [24].

An influence of Mtln on kidney function was predicted based on differential gene expression analysis. The *Mtln* gene was found to be up-regulated in human fibrotic kidney disease [25], while the *Mtln* knockout protected the kidney from fibrosis at a number of damaging conditions [25]. 

In this work, we set up to investigate kidney-related phenotypes of the Δ*Mtln*-1 knockout mice line we created earlier [16,20]. Unlike Li and co-authors [25], we haven’t performed any damaging interventions but rather observed kidney function in the aged *Mtln* knockout mice. 

## 2. Results

### 2.1. Kidney Morphology Changes upon Mtln Inactivation

This study of *Mtln’s* physiological function was started with an accidental observation. While maintaining Δ*Mtln*-1 knockout mice for over a year, we noticed a reduced lifetime of the male mice with the gene knockout relative to that of the corresponding wild-type mice. An 18-month-old knockout male mouse demonstrating the signs of immobility, recumbency, rough hair coat, hunched posture, and a weak reaction to external stimuli was euthanized and used for a complete histologic analysis to identify the cause of pathology. As a result, signs of kidney malfunction were observed (Supplementary Figure S1a).

**Figure 1:**
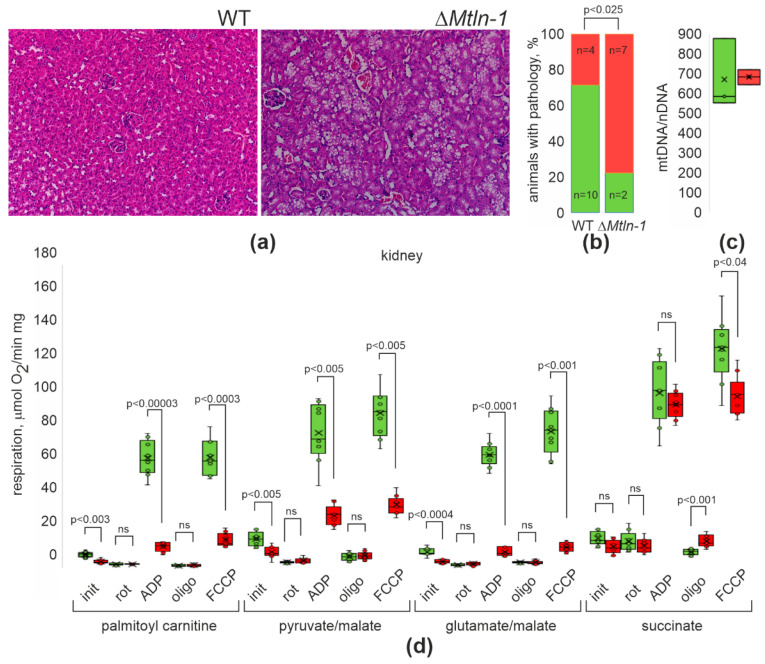
Mtln influence on kidney structure and respiration. (**a**) Hematoxylin/eosin staining of kidney samples from the wild-type (left panel) and Δ*Mtln*-1 (right panel) 12 months old male mice. Vacuolar degeneration of proximal channels is visible on the panel corresponding to the Mtln deficient mouse (right). (**b**) Frequency of the proximal channels degeneration manifestation (red bars correspond to the pathology, green bars to the lack of pathology) in the wild-type (left bars, *n* = 14) and Δ*Mtln*-1 (right bars, *n* = 9) male mice, 12 months old. (**c**) Number of mitochondria as addressed by quantitative PCR of mtDNA (*mtND1* gene) vs. nuclear DNA (*Ndufv1* gene) from the wild-type (green bars, *n* = 3 biological replicates, 3 technical replicates each) and Δ*Mtln*-1 (red bars, *n* = 3 biological replicates, 3 technical replicates each) mice. (**d**) Oxygen consumption rate (OCR) of kidney mitochondria extracted from the wild-type (green bars, *n* = 3 biological replicates, 3 technical replicates each) and Δ*Mtln*-1 (red bars, *n* = 3 biological replicates, 3 technical replicates each) mice. The groups of bars correspond to the respiration on palmitoyl carnitine (CI + CII + ETF activity), pyruvate and malate (CI activity), glutamate and malate (CI activity), and succinate (CII activity) as marked below the graphs. The experimental points measured are substrates alone (init), substrates with rotenone (rot), substrates and ADP (ADP), substrates, ADP and oligomycin (oligo), substrates and FCCP (FCCP). For panels (**b**,**c**), interquartile ranges are shown as solid bars, while all data range by thin lines. Horizontal line corresponds to the median, the cross to the average. Significance level calculated accordingly to the Student’s *t*-test is shown.

To find the tissue most affected by Δ*Mtln*-1 knockout, we performed a histological analysis of yearling knockout and wild-type male mice tissues using an aged mice population. While we found very few pathological differences overall, as we described in the preceding publication [20], we specifically detected a vacuolar degeneration of kidney proximal channels (Figure 1a) in the majority of knockout mice analyzed, while fewer wild-type mice demonstrated similar pathology (Figure 1b). Histopathological analysis of the kidneys of younger, 6-month-old male mice (Supplementary Figure S1b) or aged, 24-month-old female mice, has not revealed similar pathology. 

### 2.2. Respiration of Kidney Mitochondria upon Mtln Inactivation

An influence of Mtln gene functionality on respiration efficiency was repeatedly observed on both cellular [12,13,14,15] and mice [13,14,20] models. The kidney relies on oxidative phosphorylation as a major energy source. The quantity of mitochondria in the kidney has not changed upon *Mtln* inactivation as addressed by mtDNA (Figure 1c) quantitation. To evaluate the influence of Mtln on renal mitochondria function, we measured mitochondrial respiration using palmitoyl carnitine (Figure 1d, 1st group of bars), glutamate/malate (Figure 1d, 2nd group of bars), pyruvate and malate (Figure 1d, 3rd group of bars) and succinate (Figure 1d, 4th group of bars). The choice of substances is dictated by known controversies in the role of Mtln in the complex I-dependent respiration on fatty acid vs. carbohydrate-derived oxidative substrates [12,13,15,18,20]. We observed a decreased Complex I-dependent respiration for kidney mitochondria devoid of Mtln. The difference between the wild-type kidney mitochondria and the Δ*Mtln-1* knockout mice was observed for all respiratory Complex I (CI) substrates, but not succinate, which is the substrate for respiratory Complex II (CII). Likewise, respiration stimulated by the excess of ADP (Figure 1d, 2nd group of bars) and respiration uncoupled from ATP production (Figure 1d, rightmost bars) was inhibited by inactivation of *Mtln*, reminiscent of our earlier observations on the cell culture *Mtln* knockouts [12] and muscle mitochondria of *Mtln* knockout mice [20]. 

### 2.3. Cardiolipin Damage in Kidney Mitochondria upon Mtln Inactivation

**Figure 2:**
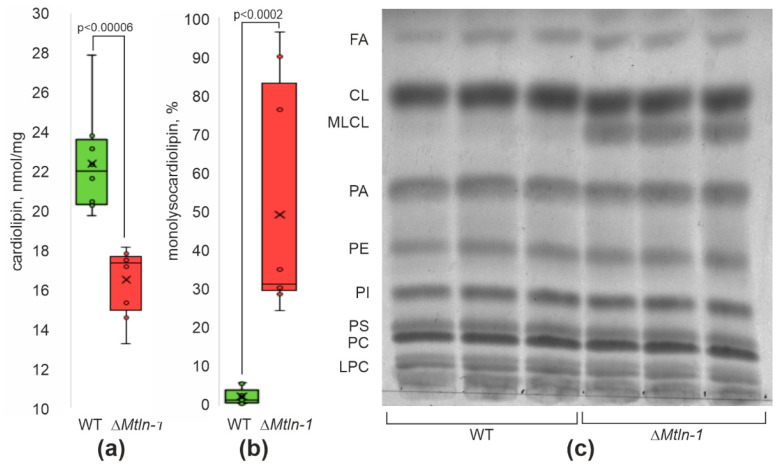
Mtln influences cardiolipin homeostasis in kidney mitochondria. (**a**) Cardiolipin quantitation in the kidney mitochondria of the wild-type male (green bars, *n* = 8) and Δ*Mtln*-1 male (red bars, *n* = 9) mice. (**b**) Quantitation of the amount of monolysocardiolipin (MLCL) relative to the total amount of cardiolipin and monolysocardiolipin (MLCL + CL) in the kidney mitochondria of the wild-type male (green bars, *n* = 9) and Δ*Mtln*-1 male (red bars, *n* = 9) mice. (**c**) Thin layer chromatography of the kidney mitochondrial lipids of the wild-type (left 3 lanes) and Δ*Mtln*-1 (right 3 lanes) mice. Lipid designations are fatty acids (FA), cardiolipin (CL), monolysocardiolipin (MLCL), phosphatidic acid (PA), phosphatidylethanolamine (PE), phosphatidylinositol (PI), phosphatidylserine (PS), phosphatidylcholine (PC), and lysophosphatidylcholine (LPC). For panels (**a**,**b**), interquartile ranges are shown as solid bars, while all data range by thin lines. Horizontal line corresponds to the median, the cross to the average. Significance level calculated accordingly to the Student’s *t*-test is shown.

Earlier, we observed a decrease in cardiolipin amount and an increase in monolysocardiolipin in muscle mitochondria of mice devoid of Mtln [20], likely explained by a predominance of cardiolipin damage over cardiolipin repair. Since cardiolipin is an ubiquitous mitochondria-specific lipid present in the organelles of all tissues, it was logical to assume that kidney mitochondrial cardiolipin might be affected by *Mtln* knockout similar to that of cardiolipin of muscles. To this end, we tested whether kidney mitochondria of *Mtln* knockout mice are depleted with cardiolipin (CL) and possess an increased amount of monolysocardiolipin (MLCL) similar to that observed earlier for muscle mitochondria. Consistent with our observations for soleus and tibialis anterior, we detected a decrease in the amount of CL (Figure 2a) and an increase in the proportion of MLCL (Figure 2b,c).

### 2.4. Kidney Mitochondrial Creatine Kinase Oligomerization and Activity Depend on Mtln Functionality

In our previous experiments with oxidative soleus and glycolytic tibialis anterior muscles of the Δ*Mtln*-1 knockout mice, we observed an influence of Mtln on the oligomerization and activity of the mitochondrial creatine kinase (mtCK) [20]. This influence is likely to be caused by the excessive destruction of CL. Although the creatine (Cr) shuttle is less important for the kidney, we assayed mtCK oligomerization (Supplementary Figure S2a) and activity (Supplementary Figure S2b) similar to that of muscle mitochondria. We observed an inhibition of mtCK octamerization and a decrease in the mtCK activity in the kidney mitochondria of Δ*Mtln*-1 mice.

An assay on the respiration coupled to ADP regeneration via Cr phosphorylation (Supplementary Figure S2c) demonstrated a general inhibition of CI-dependent respiration irrespective of the Cr shuttle in agreement with less importance of the Cr/CrP shuttling of kidney cells. 

### 2.5. Reduction of Glomerular Filtration Rates upon Mtln Inactivation

**Figure 3:**
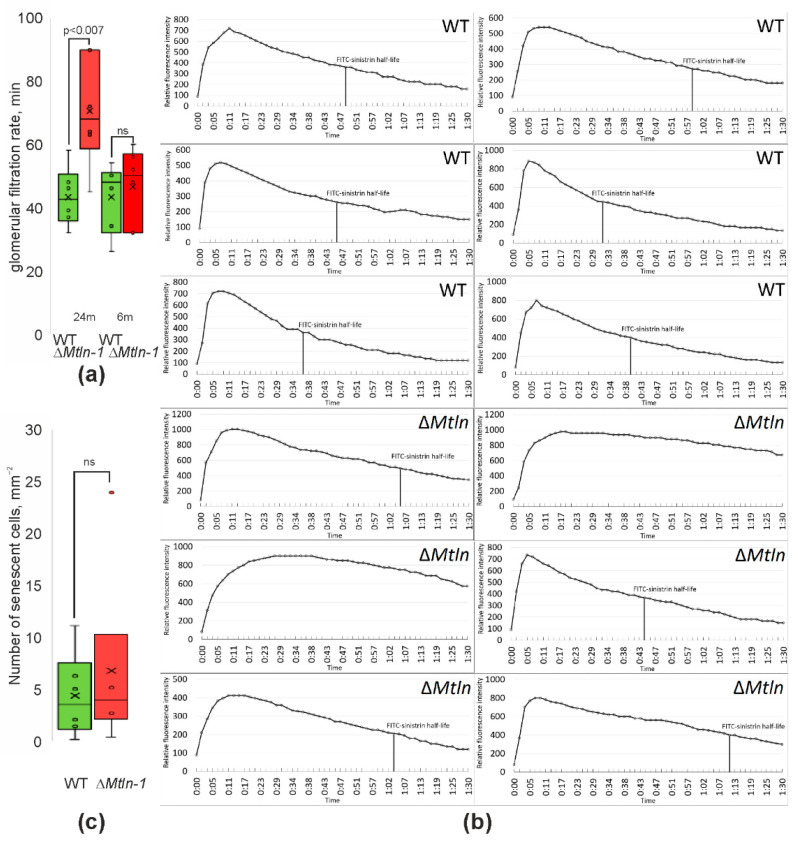
Mtln influences kidney functionality. (**a**) Glomerular filtration rates for the wild-type (green bars, left side, *n* = 7) and Δ*Mtln*-1 (red bars, left side, *n* = 6) female 24 months old mice and wild-type (green bars, right side, *n* = 6) and Δ*Mtln*-1 (red bars, right side, *n* = 6) male 6 months old mice. Values correspond to the half-periods of FITC-sinistrin blood clearance assessed by transdermal fluorescence monitor. (**b**) Primary data on glomerular filtration rate (GFR). Shown are transdermally measured fluorescence intensity curves indicating FITC-sinistrin clearance from the bloodstream. (**c**) Quantitation of senescent cell number per mm^2^ in the kidney of the wild-type (green bars, *n* = 7) and Δ*Mtln*-1 (red bars, *n* = 6) 24 months old female mice. ns-not significant. For panels (**a**,**c**), interquartile ranges are shown as solid bars, while all data range by thin lines. Horizontal line corresponds to the median, the cross to the average. Significance level calculated accordingly to the Student’s *t*-test is shown.

Histological analysis, as well as mitochondrial cardiolipin level or respiration, does not directly demonstrate an impairment in kidney function. To check whether *Mtln* inactivation would affect kidney performance, we determined glomerular filtration rates (GFR) via transdermal monitoring of the FITC-sinistrin clearance from the bloodstream [26]. In this experiment, mice were injected with fluorescently labeled sinistrin, normally cleared by renal filtration. Fluorescence intensity decay in the blood was conveniently monitored by a miniature fluorimeter mounted on the skin of mice after fur shaving. While an absolute value of the fluorescence is uninformative due to the variation in the blood vessel networks below the monitor, the speed of the fluorescence decrease is indicative of the glomerular filtration rate. While young (6-month-old) male mice demonstrated no dependence of GFR on the Mtln functionality, we observed a significant deceleration of glomerular filtration for aged (24-month-old) female Δ*Mtln-1* mice (Figure 3a,b).

The suboptimal kidney function of the aged Δ*Mtln*-1 mice might be explained by an excessive accumulation of senescent cells. To this end, we stained kidney slices of the 24-month-old female mice for β-galactosidase positive cells and counted senescent cells (Figure 3c). However, no significant difference in the density of senescent cells between the wild-type and Δ*Mtln*-1 mice was found.

### 2.6. Mtln Inactivation Perturbs Kidney Gene Expression

**Figure 4:**
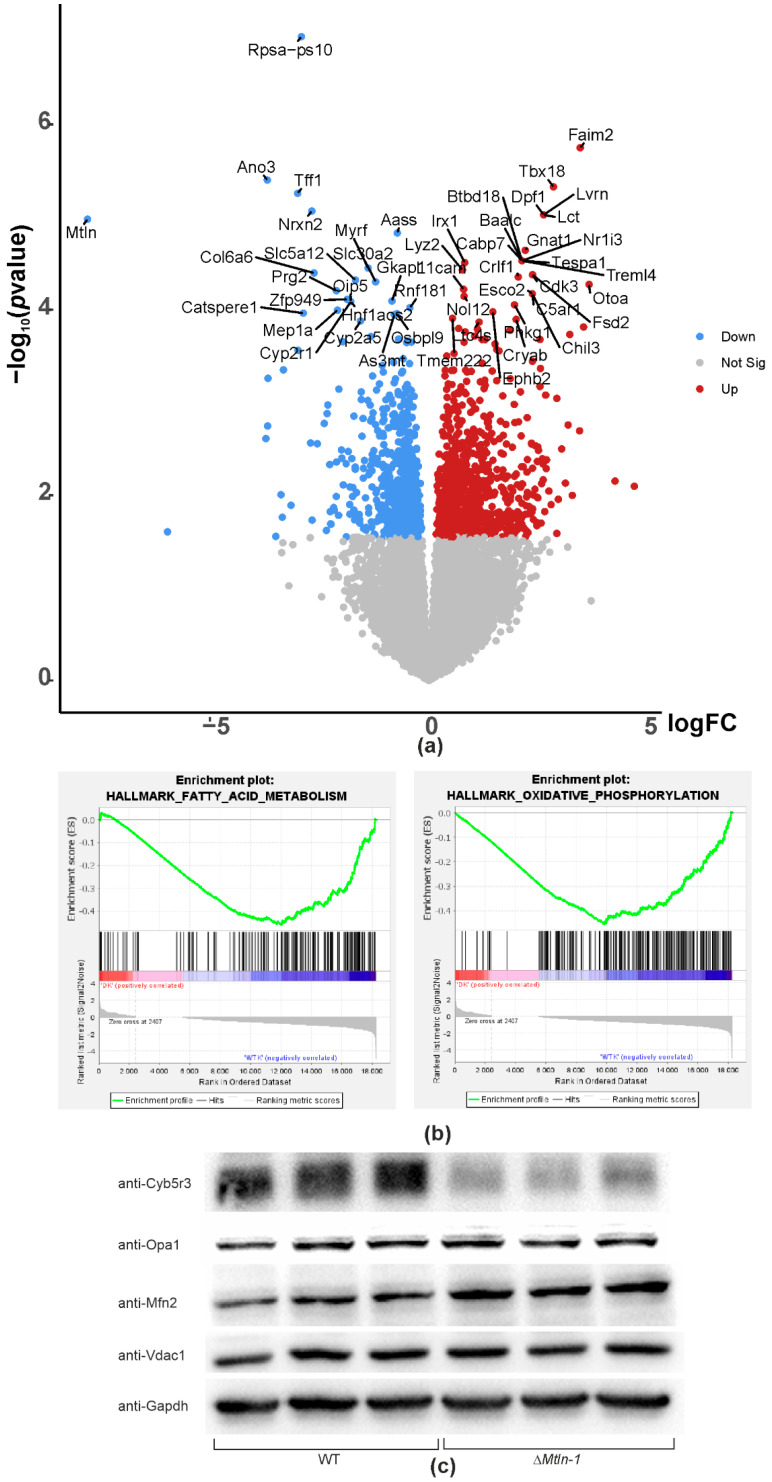
Differential gene expression in the kidney of the wild-type and Δ*Mtln*-1 knockout mice. (**a**) Volcano plot of differentially expressed genes. X-axis corresponds to the log-scale fold change of expression, Δ*Mtln*-1, relative to the wild-type, while the y-axis corresponds to the *p*-value. (**b**) Gene set enrichment analysis result demonstrating downregulation of genes from the category of fatty acids metabolism (left panel) and the category of oxidative phosphorylation (right panel) in the kidneys of Δ*Mtln*-1 mice. (**c**) Immunoblotting of kidney extracts of the wild-type (left 3 lanes) and Δ*Mtln*-1 (right 3 lanes) mice. Antibodies against mitochondrial proteins Cyb5r3, Opa1, Mfn2, Vdac1, and control antibodies against Gapdh were used as indicated left to the panels.

To get further insight into the functional role of Mtln in the kidney, we performed sequencing of kidney transcriptome for the Δ*Mtln*-1 mice (Supplementary Table S1, Figure 4a). 

Unlike muscle transcriptome [20], which demonstrated a minor difference overall, the kidney demonstrated more genes whose expression was significantly up or downregulated. Gene set enrichment analysis (GSEA) [27] of the differentially expressed genes resulted in a finding of the following top-ranked categories that were enriched in the downregulated genes: fatty acids metabolism (Figure 4b, left panel), bile acids metabolism, protein secretion, xenobiotic metabolism, Myc targets, and oxidative phosphorylation (Figure 4b, right panel).

Immunoblotting of the kidney extracts of *Mtln* knockout mice (Figure 4c, Supplementary Figure S3) corroborated a previously obtained result by mtDNA qPCR, namely, that the number of mitochondria is unchanged upon *Mtln* inactivation. A moderate increase in the amount of mitofusin (Mfn2) was observed, which could not be explained by excessive transcription (Supplementary Table S1), but most likely by protein stabilization. Mfn2 is a protein whose stability is controlled by PARK2/PINK1-dependent degradation [28], which might be downregulated upon *Mtln* inactivation. 

In our previous study [12], we observed an interaction of Mtln with Cyb5r3, an oxidoreductase involved in fatty acids desaturation [29], and several other biosynthetic processes related to hydrophobic compounds [30]. Often, protein partners stabilize each other against possible unfolding and proteolytic degradation. To address this issue, we performed an immunoblotting of the Δ*Mtln*-1 kidney extracts with anti-Cyb5r3 antibodies (Figure 4c, panel anti-Cyb5r3, Supplementary Figure S3). A drastic decrease in the amount of Cyb5r5 upon *Mtln* inactivation was observed.

## 3. Discussion

Small mitochondrial protein Mtln is involved in respiration mediated by the CI complex [12,20] and regulation of fatty acids metabolism [12,13,14,16]. An involvement of Mtln in muscle [13,14,20] and adipose tissue [15,16] functioning is documented. Here we addressed the role of this small protein in the functioning of kidneys. We revealed in this study that vacuolar degeneration of kidney proximal channels caused by *Mtln* gene knockout poses a life threat. The kidney is the second most oxygen-consuming organ [31], whose proximal tubule cells consume a major share of the oxygen to produce ATP fueling an array of transmembrane solute pumps. Multiple mitochondrial diseases have specific renal manifestations [21,32]. Symptoms observed earlier for *Mtln* knockout mice [16], such as elevated serum concentrations of lactate and triglycerides accompanied by increased body mass, are associated with type II diabetes [33]. Mitochondrial dysfunction contributes to the progression and complications of this disease [31,34]. The role of mitochondrial peptides, such as Humanin and MOTS-c, in kidney function has been previously reported [24]. Moreover, kidney-related Mtln function was also reported in a recently published manuscript [25]. An increase in *Mtln* expression was found to accompany chronic kidney disease leading to extracellular matrix deposition substituting normal kidney tissue. Artificial reduction of *Mtln* expression leads to reduced kidney fibrosis upon unilateral ureteral obstruction [25]. In our study, we observed detrimental age-associated kidney damage upon *Mtln* inactivation. The manifestation of the pathology and a disease model in our study and that of Li and co-authors [25] are different. It is unlikely that Mtln functionality would be retained in evolution if it were dangerous for kidney function. On the contrary, it is likely that an increase in *Mtln* expression regarding kidney damage is a secondary effect aiming to compensate for renal dysfunction.

In this study, we observed two age- and gender-dependent kidney pathologies associated with a loss of Mtln function, vacuolar degeneration of kidney proximal channels in aged males, and a deceleration of the glomerular filtration in aged females. Younger mice did not display these pathologies, likely because the destructive effect of Mtln loss on mitochondria accumulates with age. The aging-specific phenotype of mitochondrial peptide inactivation is common. For a recent review on the association of mitochondrial peptides and aging, see [35]. Gender-specific differences in kidney pathology related to mitochondria function are also known (see [36] for a review). In the current study, we revealed the difference in age-related kidney malfunction in male and female mice devoid of Mtln. Further work is needed to uncover molecular mechanisms leading to the age- and gender-specificity of *Mtln* knockout renal phenotypes. 

While molecular mechanisms linking Mtln peptide with the phenotypic manifestations of its knockout are still enigmatic, our study contributes to the understanding of this process. An observed decrease in the concentration of Cyb5r3, an Mtln partner protein, upon *Mtln* inactivation, might also contribute to an observed phenotype. Accumulation of MLCL at the expense of CL concentration decrease indicates CL damage, which is excessive over CL repair. It could be hypothesized that this damage, exacerbated with age, might cause mitochondrial dysfunction leading to kidney malfunction. 

## 4. Materials and Methods

### 4.1. Mice Housing and Breeding

All manipulations were conducted in compliance with the protocol approved by the Local Bioethics Commission of the Research Center “Institute of Mitoengineering of Moscow State University” LLC, (Moscow, Russia). Commission decision #79 dated July 2015, #133 dated 23 April 2018, the Bioethics Commission of Lomonosov MSU #76 dated 10 May 2018.

*Mtln* knockout mice were generated as described [16]. The animals were kept in individually ventilated cages (IVC system, TECNIPLAST S.p.A., Buguggiate, Italy), with unrestricted access to food and water, in an environment free of specific pathogens, under a 12:12 h light/dark cycle, 35 lux. Founder pups and their descendants mated with inbred C57BL/6J mice were genotyped by genomic DNA amplification with the primers GAGTCAGGGAACTCTGCTTCCTTT and CTCAGGCCAGGTCCAGCTTTTTC, followed by Sanger sequencing (Center of Collective Use «Genome» at Engelhard Institute of Molecular Biology, Moscow, Russia).

Experiments were performed on 12- and 6-month-old males and 24-month-old females, as indicated in the text. 

### 4.2. Glomerular Filtration Rate

Intravital study of kidney functional activity was carried out using a miniature fluorescent detector (MediBeacon, Mannheim, Germany) attached directly to the skin on the back of the animal. It measured the excretion kinetics of the exogenous GFR tracer, fluorescein-isothiocyanate (FITC) conjugated sinistrin [26]. In contrast to the standard protocol, in our research, animals in the experiment were under general injection anesthesia (a combination of Zoletil and Xylazine). The injection of 40 mg/mL FITC-sinistrin (in PBS) at a dose of 0.15 mg/g was carried out in the retroorbital sinus. The collected data were obtained from a transdermal sensor in the MB Lab ver.2.26 program (MediBeacon, Mannheim, Germany). According to a standard protocol, we compared the FITC-sinistrin half-life excretion time by relative fluorescence intensity [26].

### 4.3. Histology

Male and female kidneys were used for histopathological examination. The specimens were fixed with 10% buffered formalin solution (pH 7.4), trimmed, dehydrated with 99.7% isopropanol, and paraffin-embedded. Microtome sections (3 µm) were deparaffinized, hydrated, and stained with hematoxylin and eosin. Pathologies were diagnosed and classified according to published recommendations [37,38].

To stain beta-galactosidase-positive senescent cells, we used the protocol for paraffin sections created by V.N. Manskikh [39]; kidney samples were fixed with Baker’s formol-calcium solution (1 g CaCl_2_, 10 mL 37% formaldehyde, 90 mL water) [40] during 24 h at +4 °C, washed in distilled water for 24 h, dehydrated in 3 changes of absolute acetone at +4 °C for 2 h in each, cleared 1 h in benzene and embedded in paraffin with Z. Lojda et al. method for enzyme histochemistry [41]. Microtome sections with a thickness of 5 µm were stained 3 h with X-gal solution (20 mg X-gal, 1 mL Dimethylformamide, 5 mL 1.65% K_3_[Fe(CN)_6_], 5 mL 2.11% K_4_[Fe(CN)_6_], 70 mL 0.1 M citrate buffer pH 6.0) at suboptimal pH 6.0 and 37 °C as recommended [42] and counterstained with nuclear red. The number of senescent cells in the full organ section were counted manually and normalized to 1 mm^2^ of section area with ImageJ v1.4.3 software on organ photomicrographs.

### 4.4. Tissue Processing, Mitochondria Preparation, and Respiration Analysis

Kidneys were separated from the capsule upon extraction and placed in an ice-cold isolation medium. Part of the kidney (5–10 mg) was immediately frozen in liquid nitrogen and stored at −80 °C for later determinations of enzyme activities, oligomer state of creatine kinase, and creatine concentration. Mitochondria preparation from kidneys of wild-type and mutant mice was done as described before with slight modifications [43]. For that, tissue fragments were gently minced by small cooled scissors into 0.5 mm pieces and, due to the small size of samples, homogenized in a homemade 0.5 mL Potter Teflon–glass microhomogenizer with 200-micron clearance in 10 volumes (*v*/*w*) of 250 mM sucrose, 0.5 mM EGTA, 20 mM HEPES-NaOH, pH 7.6, and 0.1% BSA (isolation medium) for 2 min at 4 °C, with a ratio of 5/1 volume to weight of tissue fragment. The homogenate was centrifuged at 1000× *g* for 10 min at 4 °C in a centrifuge (Eppendorf, Hamburg, Germany). The supernatant was collected and centrifuged at 9000× *g* under the same conditions. Mitochondrial pellets were collected and suspended in the same volume of isolation medium lacking BSA (a microhomogenizer and centrifugation at 10,500 *g* for 10 min at 4 °C were used). The resulting pellet was suspended in a minimal volume (approximately 1 uL/mg of initial tissue weight) with a typical concentration of 90–100 mg/mL, as determined using the bicinchoninic acid method with BSA as the standard, according to the manufacturer’s instructions (Pierce, Waltham, MA, USA). All procedures were performed in a cold box at 4 °C.

To assess the respiration capacity of the isolated mitochondria, the rate of oxygen consumption was measured at 25 °C using a closed-type Clark electrode on Hansatech oxygraph (Narborough, UK) as described before [43]. Mitochondria (0.05–0.1 mg protein) were incubated in an oxygraph cell containing 0.5 mL of MIR05 [44] respiration medium (EGTA 0.5 mM, 3 mM MgCl_2_, 60 mM potassium lactobionate, 20 mM taurine, 10 mM KH_2_PO_4_, 20 mM HEPES, 110 mM sucrose, 1 g/L BSA) and the efficiency of respiration was evaluated in the presence of 5 mM glutamate/1.25 mM malate, 5 mM pyruvate/1 mM malate or 5 mM succinate/ 2 mM rotenone or 10 µM palmitoyl-L-carnitine. Then indicated 1 µM oligomycin, or 0.1 mM ADP or 0.1 mM ATP/2 mM creatine, or 10 nM FCCP was added. 

### 4.5. Lipid Analysis

Cardiolipin content in isolated mitochondria was estimated using the Cardiolipin Assay Kit (ab241036, Abcam, Waltham, MA, USA) according to manufacture protocol.

Lipids extraction was performed according to the Bligh and Dyer method under a stream of nitrogen with oxygen-free solutions bubbled with N_2_ [45]. Extracted lipids were solved in a chloroform/methanol mixture 2:1 (*v*/*v*). Thin-layer chromatography was done according to the published procedure [46]. Analytical grade organic solvents and HPTLC chromatography plates (20 × 10 cm silica gel 60 F254 aluminum plates) were obtained from E. Merck (Darmstadt, Germany). Before sample or standard application, HPTLC plates were prepared by immersion in 2.3% boric acid in ethanol and then dried and activated at 110 °C for 20 min.

Samples were applied with the homemade glass capillary sample applicator with valve (driven by nitrogen stream) as 10 mm-long bands, 15 mm from the bottom of the plate, at a constant application rate of about 200 nL/s, under continuous drying with a stream of nitrogen at 4 bars. For PLs standards (Avanti Polar Lipids, Alabaster, AL, USA), a stock solution (1 mg/mL) was prepared in chloroform/methanol (2:1, *v*/*v*).

Elution was done in a glass chamber equilibrated with a vapor of eluent for 1 h. The eluent consisted of a mixture of chloroform/ethanol/triethylamine/water (3/3.5/3.5/0.7, *v*/*v*).

After a 1D development, drying, and immersion in the staining reagent bath with 0.5% copper sulfate (*w*/*v*) in 1.16 m orthophosphoric acid (2 min), plates were dried at fume hood for 2 h at room temperature and heated at sand bath for 15 min at 155 °C to carbonize organic matter and visualize PLs.

Plates were photographed in reflection mode under white light in the Biorad ChemiDoc. Images were analyzed in absorbance mode. PL spot intensities were integrated, and peaks surfaces were expressed in arbitrary units with the help of Image J and, after calibration with CL and MLCL standards, converted to nmoles/mg of mitochondrial protein.

### 4.6. Whole Transcriptome Assay

Transcriptome libraries were prepared using mRNA isolated by polyA fractionation and Dynabeads^®^ mRNA DIRECT™ Micro Kit (Invitrogen, Waltham, MA, USA) from kidney tissue of wild-type (*n* = 3) and *Mtln* knockout (*n* = 3) mice. The quality and concentrations of libraries were measured by the automated electrophoresis system Agilent 4200 Bioanalyzer^TM^ with the High Sensitivity ScreenTape kits (Agilent, Santa Clara, CA, USA) and Qubit 4 fluorimeter (Thermo Fisher Scientific, Waltham, MA, USA), respectively. The libraries proceeded in three replicas using an Ion Total-RNA Seq Kit v2 according to the manual provided. Chip loading was done using Ion Chef™ Instrument with the Ion 540™ Chef Kit (Thermo Fisher Scientific, USA), while sequencing was performed on Ion GeneStudio™ S5 System (Thermo Fisher Scientific, USA).

Read quality was analyzed by the FastQC program. Reads with unsatisfactory quality and/or length were removed utilizing the Trimmomatic-0.36 package. The reads were aligned from the obtained files to the reference genome of *Mus musculus* with the assembly GRCm39 (GCA_000001635.9) by the STAR 2.7 software [47]. The resulting files with reads aligned and sorted by coordinates were used to obtain the count matrix using the HTSeq package. The obtained count matrix was analyzed using the web application Phantasus, integrated into the R environment. Differentially expressed genes were evaluated using the Limma package. Adjustment *p*-values (q-value or FDR, false discovery rate) for genes were set at less than 0.05 to detect differentially expressed ones. Functional enrichment analysis was performed with GSEA [27] against h.all.v7.5.symbols database.

### 4.7. Immunoblotting

Immunoblotting of tissue lysates was done as previously described [39]. For immunoblot analysis, samples of kidney tissue were lysed in RIPA buffer (150 mM sodium chloride, 50 mM Tris-HCl pH 8.0, 0.5% Nonidet P-40, 1% sodium deoxycholate, 0.5% SDS) with protease inhibitor cocktail (ThermoFisher Scientific, Waltham, MA, USA). The transfer-ready PVDF membrane (Thermo Scientific, Waltham, MA, USA) was blocked for 1 h in TBST (10 mM Tris-HCl pH 7.5, 150 mM NaCl, 0.1% Tween-20) containing 5% bovine serum albumin (BSA, Proliant Biologicals, Ankeny, IA, USA). All primary antibodies were diluted 1:1000 in TBST containing 5% BSA. The following primary antibodies were used: anti-OPA1 (ab42364, Abcam, Waltham, MA, USA), anti-MFN2 (ab56889, Abcam, Waltham, MA, USA), anti-CYB5R3 (sc-398043), anti-VDAC1 (ab15895, Abcam, Waltham, MA, USA). 

The secondary HRP-conjugated anti-mouse (1721011, Biorad, Hercules, CA, USA) and anti-rabbit (1706515, Biorad, Hercules, CA, USA) antibodies were used at a 1:3000 dilution. GAPDH (ab8245, Abcam, Waltham, MA, USA) was used as a loading control. 

### 4.8. qPCR

Assessment of the mitochondrial-to-nuclear DNA ratio was done as previously described [39]. DNA was extracted from 5–15 mg of kidney tissue with GeneJET Genomic DNA Purification Kit (ThermoFisher Scientific, Waltham, MA, USA). Quantitative PCR gene amplifications were performed using SYBR^®^ Green PCR master mix (ThermoFisher Scientific, Waltham, MA, USA) in the CFX384 Touch Real-Time PCR System. qPCR was conducted using primer sets: mt-Nd1 5′-TCCCCTACCAATACCACACC-3′, 5′-CGGCTCGTAAAGCTCCGAAT-3′, and Ndufv1 5′-GATGTGTTTGTGGTGCGTGG-3′, 5′-GAATTGCGTTCTCGGCCAAA-3′. The amount of mitochondrial DNA was calculated by the 2−*ΔΔ*CT method and normalized to the nuclear DNA.

## Data Availability

Results of transcriptome sequencing are available in SRA via accession PRJNA844239.

## Supplementary Materials

The following supporting information can be downloaded at: https://www.mdpi.com/article/10.3390/ijms24109106/s1.

## References

[1] Ruiz-Orera J., Albà M.M. (2019). Translation of Small Open Reading Frames: Roles in Regulation and Evolutionary Innovation. Trends Genet..

[2] Makarewich C.A., Olson E.N. (2017). Mining for Micropeptides. Trends Cell Biol..

[3] Couso J.-P., Patraquim P. (2017). Classification and Function of Small Open Reading Frames. Nat. Rev. Mol. Cell Biol..

[4] Chugunova A., Navalayeu T., Dontsova O., Sergiev P. (2018). Mining for Small Translated ORFs. J. Proteome Res..

[5] Rath S., Sharma R., Gupta R., Ast T., Chan C., Durham T.J., Goodman R.P., Grabarek Z., Haas M.E., Hung W.H.W. (2021). MitoCarta3.0: An Updated Mitochondrial Proteome Now with Sub-Organelle Localization and Pathway Annotations. Nucleic Acids Res..

[6] Rathore A., Chu Q., Tan D., Martinez T.F., Donaldson C.J., Diedrich J.K., Yates J.R., Saghatelian A. (2018). MIEF1 Microprotein Regulates Mitochondrial Translation. Biochemistry.

[7] Brown A., Rathore S., Kimanius D., Aibara S., Bai X.-C., Rorbach J., Amunts A., Ramakrishnan V. (2017). Structures of the Human Mitochondrial Ribosome in Native States of Assembly. Nat. Struct. Mol. Biol..

[8] Chen J., Brunner A.-D., Cogan J.Z., Nuñez J.K., Fields A.P., Adamson B., Itzhak D.N., Li J.Y., Mann M., Leonetti M.D. (2020). Pervasive Functional Translation of Noncanonical Human Open Reading Frames. Science.

[9] Zhang S., Reljić B., Liang C., Kerouanton B., Francisco J.C., Peh J.H., Mary C., Jagannathan N.S., Olexiouk V., Tang C. (2020). Mitochondrial Peptide BRAWNIN Is Essential for Vertebrate Respiratory Complex III Assembly. Nat. Commun..

[10] Chu Q., Martinez T.F., Novak S.W., Donaldson C.J., Tan D., Vaughan J.M., Chang T., Diedrich J.K., Andrade L., Kim A. (2019). Regulation of the ER Stress Response by a Mitochondrial Microprotein. Nat. Commun..

[11] Sergiev P.V., Rubtsova M.P. (2021). Little but Loud. The Diversity of Functions of Small Proteins and Peptides—Translational Products of Short Reading Frames. Biochem. Moscow..

[12] Chugunova A., Loseva E., Mazin P., Mitina A., Navalayeu T., Bilan D., Vishnyakova P., Marey M., Golovina A., Serebryakova M. (2019). *LINC00116* Codes for a Mitochondrial Peptide Linking Respiration and Lipid Metabolism. Proc. Natl. Acad. Sci. USA.

[13] Stein C.S., Jadiya P., Zhang X., McLendon J.M., Abouassaly G.M., Witmer N.H., Anderson E.J., Elrod J.W., Boudreau R.L. (2018). Mitoregulin: A LncRNA-Encoded Microprotein That Supports Mitochondrial Supercomplexes and Respiratory Efficiency. Cell Rep..

[14] Makarewich C.A., Baskin K.K., Munir A.Z., Bezprozvannaya S., Sharma G., Khemtong C., Shah A.M., McAnally J.R., Malloy C.R., Szweda L.I. (2018). MOXI Is a Mitochondrial Micropeptide That Enhances Fatty Acid β-Oxidation. Cell Rep..

[15] Friesen M., Warren C.R., Yu H., Toyohara T., Ding Q., Florido M.H.C., Sayre C., Pope B.D., Goff L.A., Rinn J.L. (2020). Mitoregulin Controls β-Oxidation in Human and Mouse Adipocytes. Stem Cell Rep..

[16] Averina O.A., Permyakov O.A., Emelianova M.A., Grigoryeva O.O., Gulyaev M.V., Pavlova O.S., Mariasina S.S., Frolova O.Y., Kurkina M.V., Baydakova G.V. (2023). Mitochondrial Peptide Mtln Contributes to Oxidative Metabolism in Mice. Biochimie.

[17] Choi M., Kang K.W. (2023). Mitoregulin Controls Mitochondrial Function and Stress-Adaptation Response during Early Phase of Endoplasmic Reticulum Stress in Breast Cancer Cells. Biochim. Biophys. Acta (BBA.)-Mol. Basis Dis..

[18] Lin Y.-F., Xiao M.-H., Chen H.-X., Meng Y., Zhao N., Yang L., Tang H., Wang J.-L., Liu X., Zhu Y. (2019). A Novel Mitochondrial Micropeptide MPM Enhances Mitochondrial Respiratory Activity and Promotes Myogenic Differentiation. Cell Death Dis..

[19] Wang L., Fan J., Han L., Qi H., Wang Y., Wang H., Chen S., Du L., Li S., Zhang Y. (2020). The Micropeptide LEMP Plays an Evolutionarily Conserved Role in Myogenesis. Cell Death Dis..

[20] Averina O.A., Permyakov O.A., Emelianova M.A., Grigoryeva O.O., Lovat M.L., Egorova A.E., Grinchenko A.V., Kumeiko V.V., Marey M.V., Manskikh V.N. (2023). Mitoregulin Contributes to Creatine Shuttling and Cardiolipin Protection in Mice Muscle. Int. J. Mol. Sci..

[21] Galvan D.L., Green N.H., Danesh F.R. (2017). The Hallmarks of Mitochondrial Dysfunction in Chronic Kidney Disease. Kidney Int..

[22] O’Toole J. (2014). Renal Manifestations of Genetic Mitochondrial Disease. Int. J. Nephrol. Renov. Dis..

[23] Finsterer J., Scorza F. (2017). Renal Manifestations of Primary Mitochondrial Disorders. Biomed. Rep..

[24] Liu C., Gidlund E.-K., Witasp A., Qureshi A.R., Söderberg M., Thorell A., Nader G.A., Barany P., Stenvinkel P., Von Walden F. (2019). Reduced Skeletal Muscle Expression of Mitochondrial-Derived Peptides Humanin and MOTS-C and Nrf2 in Chronic Kidney Disease. Am. J. Physiol.-Ren. Physiol..

[25] Li J., Qu X., Guan C., Luo N., Chen H., Li A., Zhuang H., Yang J., Diao H., Zeng S. (2023). Mitochondrial Micropeptide MOXI Promotes Fibrotic Gene Transcription by Translocation to the Nucleus and Bridging N-Acetyltransferase 14 with Transcription Factor c-Jun. Kidney Int..

[26] Scarfe L., Schock-Kusch D., Ressel L., Friedemann J., Shulhevich Y., Murray P., Wilm B., de Caestecker M. (2018). Transdermal Measurement of Glomerular Filtration Rate in Mice. JoVE.

[27] Subramanian A., Tamayo P., Mootha V.K., Mukherjee S., Ebert B.L., Gillette M.A., Paulovich A., Pomeroy S.L., Golub T.R., Lander E.S. (2005). Gene Set Enrichment Analysis: A Knowledge-Based Approach for Interpreting Genome-Wide Expression Profiles. Proc. Natl. Acad. Sci. USA.

[28] Leboucher G.P., Tsai Y.C., Yang M., Shaw K.C., Zhou M., Veenstra T.D., Glickman M.H., Weissman A.M. (2012). Stress-Induced Phosphorylation and Proteasomal Degradation of Mitofusin 2 Facilitates Mitochondrial Fragmentation and Apoptosis. Mol. Cell.

[29] Keyes S.R., Cinti D.L. (1980). Biochemical Properties of Cytochrome B5-Dependent Microsomal Fatty Acid Elongation and Identification of Products. J. Biol. Chem..

[30] Reddy V.V., Kupfer D., Caspi E. (1977). Mechanism of C-5 Double Bond Introduction in the Biosynthesis of Cholesterol by Rat Liver Microsomes. J. Biol. Chem..

[31] Forbes J.M., Thorburn D.R. (2018). Mitochondrial Dysfunction in Diabetic Kidney Disease. Nat. Rev. Nephrol..

[32] Singh H., Sheu S.-S. (2017). Pharmacology of Mitochondria.

[33] Crawford S.O., Hoogeveen R.C., Brancati F.L., Astor B.C., Ballantyne C.M., Schmidt M.I., Young J.H. (2010). Association of Blood Lactate with Type 2 Diabetes: The Atherosclerosis Risk in Communities Carotid MRI Study. Int. J. Epidemiol..

[34] Wallace D.C. (2005). A Mitochondrial Paradigm of Metabolic and Degenerative Diseases, Aging, and Cancer: A Dawn for Evolutionary Medicine. Annu. Rev. Genet..

[35] Miller B., Kim S.-J., Kumagai H., Yen K., Cohen P. (2022). Mitochondria-Derived Peptides in Aging and Healthspan. J. Clin. Investig..

[36] Sultanova R.F., Schibalski R., Yankelevich I.A., Stadler K., Ilatovskaya D.V. (2020). Sex Differences in Renal Mitochondrial Function: A Hormone-Gous Opportunity for Research. Am. J. Physiol.-Ren. Physiol..

[37] Maronpot R.R. (1999). Pathology of the Mouse: Reference and Atlas.

[38] Hard C.C., Alden C.L., Bruner R.H.G., Frith C.H., Lewis R.M., Owen R.A., Krieg K., Durchfeld-Meyer B. (1999). Non-Proliferative Lesions of the Kidney and Lower Urinary Tract in the Rat. URG-1, Guides for Toxicological Pathology.

[39] Averina O.A., Laptev I.G., Emelianova M.A., Permyakov O.A., Mariasina S.S., Nikiforova A.I., Manskikh V.N., Grigorieva O.O., Bolikhova A.K., Kalabin G.A. (2022). Mitochondrial RRNA Methylation by Mettl15 Contributes to the Exercise and Learning Capability in Mice. Int. J. Mol. Sci..

[40] Luna L. (1993). Histopathologic Methods and. Color Atlas of Special Stains and Tissue Artifacts.

[41] Lojda Z., Gossrau R., Schiebler T.H. (1979). Enzyme Histochemistry: A Laboratory Manual.

[42] Debacq-Chainiaux F., Erusalimsky J.D., Campisi J., Toussaint O. (2009). Protocols to Detect Senescence-Associated Beta-Galactosidase (SA-Βgal) Activity, a Biomarker of Senescent Cells in Culture and in Vivo. Nat. Protoc..

[43] Vyssokikh M.Y., Holtze S., Averina O.A., Lyamzaev K.G., Panteleeva A.A., Marey M.V., Zinovkin R.A., Severin F.F., Skulachev M.V., Fasel N. (2020). Mild Depolarization of the Inner Mitochondrial Membrane Is a Crucial Component of an Anti-Aging Program. Proc. Natl. Acad. Sci. USA.

[44] Gnaiger E., Kuznetsov A.V., Schneeberger S., Seiler R., Brandacher G., Steurer W., Margreiter R., Heldmaier G., Klingenspor M. (2000). Mitochondria in the Cold. Life in the Cold.

[45] Bligh E.G., Dyer W.J. (1959). A Rapid Method of Total Lipid Extraction and Purification. Can. J. Biochem. Physiol..

[46] Pinault M., Guimaraes C., Dumas J., Servais S., Chevalier S., Besson P., Goupille C. (2020). A 1D High Performance Thin Layer Chromatography Method Validated to Quantify Phospholipids Including Cardiolipin and Monolysocardiolipin from Biological Samples. Eur. J. Lipid. Sci. Technol..

[47] Dobin A., Davis C.A., Schlesinger F., Drenkow J., Zaleski C., Jha S., Batut P., Chaisson M., Gingeras T.R. (2013). STAR: Ultrafast Universal RNA-Seq Aligner. Bioinformatics.

