# Supplementary material for: Kidney-Related Function of Mitochondrial Protein Mitoregulin
Source: Int J Mol Sci. 2023 May 22;24(10):9106. doi: 10.3390/ijms24109106 (PMC10219308; doi:10.3390/ijms24109106)
Supplement: Supplementary file 1 [file ijms-24-09106-s001.zip › Averina IJMS kidney SI.pdf]

International Journal of
Molecular Sciences

MDPI

Supplementary Materials

Kidney-Related Function of Mitochondrial Protein Mitoregulin

Olga A. Averina [1,2], Oleg A. Permyakov [1], Mariia A. Emelianova [3], Ekaterina A. Guseva [3], Olga O. Grigoryeva [1], Maxim L. Lovat [2,4], Anna E. Egorova [5], Andrei V. Grinchenko [6], Vadim V. Kumeiko [5,6], Maria V. Marey [7], Vasily N. Manskikh [2,4], Olga A. Dontsova [2,3,8,9], Mikhail Y. Vyssokikh [2,7,*] and Petr V. Sergiev [1,2,3,8,*]

[1] Institute of Functional Genomics, Lomonosov Moscow State University, 119992 Moscow, Russia; averina.olga.msu@gmail.com (O.A.A.); norad_m@mail.ru (O.A.P.); grig_forever@mail.ru (O.O.G.)
[2] Belozersky Institute of Physico-Chemical Biology, Lomonosov Moscow State University, 119992 Moscow, Russia; lovat@mail.ru (M.L.L.); manskikh@mail.ru (V.N.M.); olga.a.dontsova@gmail.com (O.A.D.)
[3] Center for Life Sciences, Skolkovo Institute of Science and Technology, 143025 Moscow, Russia; mshep98@mail.ru (M.A.E.); eguseva98@mail.ru (E.A.G.)
[4] Institute of Mitoengineering MSU, 119992 Moscow, Russia
[5] Institute of Life Sciences and Biomedicine, Far Eastern Federal University, 690922 Vladivostok, Russia; bioanna95@list.ru (A.E.E.); kumeyko.vv@dvfu.ru (V.V.K.)
[6] A.V. Zhirmunsky National Scientific Center of Marine Biology, 690041 Vladivostok, Russia; grishagrin@mail.ru
[7] National Medical Research Center for Obstetrics, Gynecology and Perinatology Named after Academician V.I.Kulakov, 117198 Moscow, Russia; m_marey@oparina4.ru
[8] Department of Chemistry, Lomonosov Moscow State University, 119991 Moscow, Russia
[9] Shemyakin-Ovchinnikov Institute of Bioorganic Chemistry, Russian Academy of Sciences, 119992 Moscow, Russia
* Correspondence: mikhail.vyssokikh@gmail.com (M.Y.V.); petya@genebee.msu.ru (P.V.S.)

1. Supplementary figures

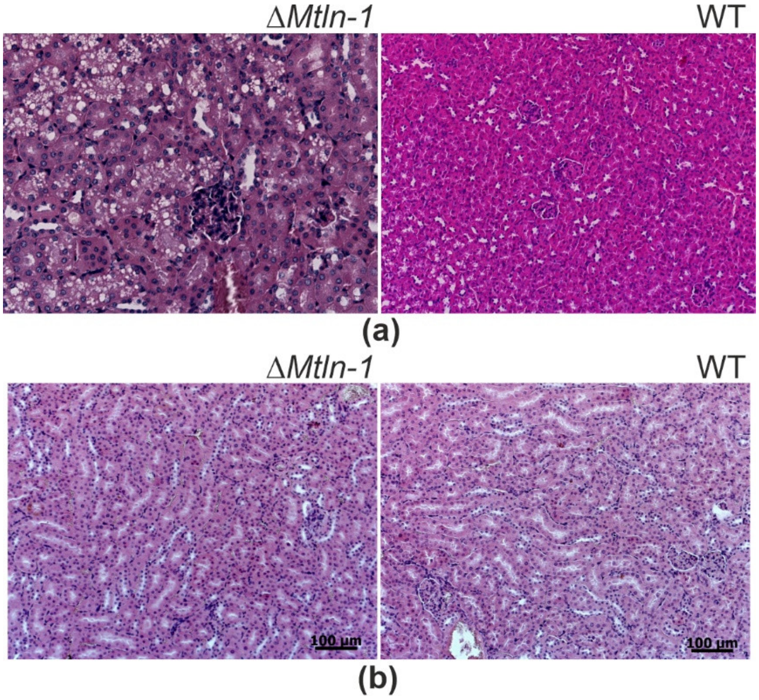

Figure S1. Mtln influence on kidney structure and metabolism. (**a**) Hematoxylin/eosin staining of kidney samples from the 18 months old wild type male (right panel) and Δ*Mtln-1* (left panel) male mice showing signs of pathology such as immobility, recumbency, rough hair coat, hunched posture, weak reaction to external stimuli (right panel). Vacuolar degeneration of proximal channels is visible on the panel corresponding to the Mtln deficient mouse (left). (**b**) Hematoxylin/eosin staining

of kidney samples from the 6 months old wild type male (right panel) and Δ*Mtln-1* (left panel) male mice. No signs of pathology are visible at this stage.

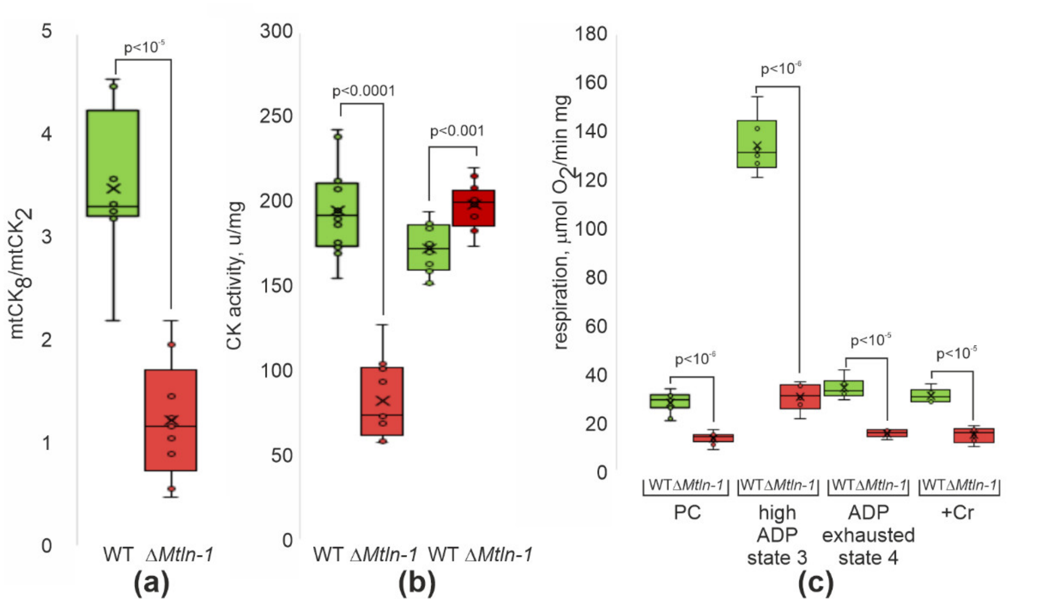

Figure S2. (**a**) Relative abundance of the octameric and dimeric forms of the mitochondrial creatine kinase (mtCK) in the kidney mitochondrial extracts of the wild type male (green bars, n=8) and Δ*Mtln* -1 male (red bars, n=9) mice. (**b**) Mitochondrial (left group of bars) and cytosolic (right group of bars) creatine kinase activity for the kidney extracts of the wild type male (green bars, n=12 samples) and Δ*Mtln-1* male (red bars, n=12 samples) mice. (**c**) OCR of kidney mitochondria extracted from the wild type male (green bars, n=6) and Δ*Mtln-1* male (red bars, n=6) mice. The first group of bars correspond to the respiration on palmitoyl carnitine only, the second group corresponds to the stimulation of the same process with excess of ADP (state 3), the third group corresponds to the ADP exhaustion (state 4), the fourth one to the following stimulation of respiration by addition of creatine.

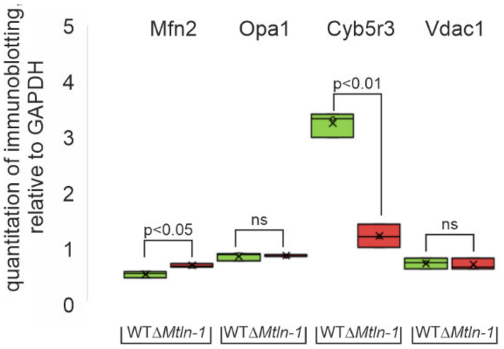

Figure S3. Quantitation of the selected mitochondrial proteins (Mfn2, Opa1, Cyb5r3 and Vdac1) indicated above the bars by immunoblotting. Kidney extracts from the wild type male (green bars, n=3) and Δ*Mtln-1* male (red bars, n=3) mice are used for immunoblotting (Figure 4c, main text). Quantitation of the corresponding bands was done with ImageJ software and related to that of the loading control (Gapdh).
